# Changes in saliva analytes in equine acute abdominal disease: a sialochemistry approach

**DOI:** 10.1186/s12917-019-1933-6

**Published:** 2019-06-06

**Authors:** María Dolores Contreras-Aguilar, Damián Escribano, Silvia Martínez-Subiela, María Martín-Cuervo, Elsa Lamy, Fernando Tecles, Jose Joaquín Cerón

**Affiliations:** 1 Interdisciplinary Laboratory of Clinical Analysis (Interlab-UMU), Veterinary School, Campus of Excellence Mare Nostrum, University of Murcia, Campus de Espinardo s/n, 30100 Espinardo, Murcia, Spain; 2 Department of Food and Animal Science, School of Veterinary Medicine, University of Barcelona, Bellaterra, 08193 Barcelona, Spain; 3 Animal Medicine, Faculty of Veterinary Medicine of Cáceres, University of Extremadura, Av. de la Universidad S-N, 10005 Cáceres, Spain; 4 Institute of Mediterranean Agricultural and Environmental Sciences, University of Évora, Núcleo da Mitra, Apartado 94, 7006-554 Évora, Portugal

**Keywords:** Biomarkers, Colic, Horse, Saliva, Sialochemistry

## Abstract

**Background:**

The biochemical components of saliva can change in certain pathologies in horses, for example in acute abdominal disease. The aim of this study was (1) to evaluate if a panel of biochemical analytes usually used in serum can be measured in saliva of horses and (2) to study the possible changes of these biochemical analytes in saliva of horses affected by acute abdominal disease. A panel of 23 analytes was analytically validated in saliva of horses and possible changes in these analytes in a pilot study with six healthy horses and six horses with acute abdominal disease were evaluated. The analytes with significant changes were then evaluated in a larger population of 20 healthy and 37 diseased horses.

**Results:**

Seven analytes showed significant increases in the pilot study which were confirmed in the larger population. The analytes which showed significant changes, and their median fold increase and significance shown in the larger population were salivary γ-glutamyl transferase (gGT, 2.3 fold, *P* = 0.001), creatine kinase (CK, 6.2 fold, *P* < 0.001), urea (2.3 fold, *P* = 0.001), total bilirubin (2.6 fold, *P* < 0.001), total proteins (3.2 fold, *P* < 0.001), phosphorus (P, 4.5 fold, *P* < 0.001) and alpha-amylase (sAA, 8.5 fold, *P* < 0.001). Total proteins, P and sAA showed sensitivities higher than 70% at their optimal cut-off points and a specificity of 100% in differentiating between healthy horses and those with acute abdominal disease.

**Conclusions:**

A panel of 23 biochemical analytes can be measured in saliva of horses, where gGT, CK, urea, total bilirubin, total protein, P and sAA levels are raised in horses with acute abdominal disease.

**Electronic supplementary material:**

The online version of this article (10.1186/s12917-019-1933-6) contains supplementary material, which is available to authorized users.

## Background

Saliva mainly consists of water, which represents up to 99% of its total volume. In addition, studies on human and veterinary species have reported that saliva also contains compounds such as phosphorus (P), lactate, cholesterol, fatty acids, glucose, hormones such as cortisol, triglycerides, urea and uric acid [1, 2]. In addition, saliva has enzymes such as salivary alpha-amylase (sAA), lipase, carbonic anhydrase, adenosine deaminase (ADA), aspartate aminotransferase (AST), alanine aminotransferase (ALT), alkaline phosphatase (ALP), lactate dehydrogenase (LDH) and creatine kinase (CK), among others [3–8].

The composition of saliva can be affected by systemic disorders [9]. Due to this, there has been increased interest over recent years in the use of saliva as a diagnostic fluid. In addition, saliva has some advantages over blood since it is easily collected by non-invasive, non-stressful procedures [10, 11] and by non-trained personnel with a minimum of material. For these reasons, a new term in human medicine, "sialochemistry", has been created, which consists of the measurement of analytes included in the routine serum biochemistry profiles in saliva [12, 13]. In animal species, saliva analytes have been studied in different situations. For example, total esterase (TEA), cholinesterase (ChE) and ADA increase in pigs affected by an acute stress or by a pathologic condition such as lameness [4, 5]. In horses, sAA and cortisol increase in acute abdominal disease, acting as possible pain-induced stress biomarkers [3]. In addition, in a proteomic study [14], acute phase proteins in saliva were observed to change in horses with this disease. However, to the authors’ knowledge, other routine biochemistry analytes have not been measured in saliva in horses with acute abdominal disease, so their potential utility as biomarkers is unknown.

The objective of this paper was to evaluate if a panel of biochemical analytes usually used in serum can be measured in saliva of horses, and also to study the possible changes of these biochemical analytes in saliva of horses affected by acute abdominal disease. For this purpose, a pilot exploratory evaluation was first made of healthy horses and those with acute abdominal disease to assess possible differences between the two groups of animals in a profile of 23 biochemical analytes. Then, those salivary analytes that significantly varied in the pilot study were later clinically validated in a larger population.

## Methods

### Pilot exploratory study

The diseased population (*n* = 6), diagnosed as having acute gastrointestinal disease, came from the Veterinary Teaching Hospital of the University of Extremadura (Table 1). The diagnosis was based on history, physical examination, haematology and plasma biochemistry (Olympus Diagnostica GmbH AU 600, Beckman Coulter, Ennis, Ireland), transabdominal ultrasonography, and response to treatment by a board-certified internist (MMC). In horses undergoing surgery, the diagnosis of the acute gastrointestinal disease was based on laparotomy findings.

**Table 1:** Overview of horses included in the pilot exploratory study

Group	Age (year)	Gender	Breed	Diagnosis
Diseased horses	23	Stallion	Pure Spanish horse	Enteritis
	4	Mare	Crossbred	Impaction of the pelvic flexure
	8	Stallion	Arabian	Impaction of the right dorsal colon
	5	Stallion	Pure Spanish horse	Strangulated lesion in the small intestine
	10	Mare	Crossbred	Fecaloma with ischemia
	7	Gelding	Crossbred	Large colon displacement
Healthy horses	5	Stallion	Pure Spanish horse	Healthy horse
	5	Stallion	Pure Spanish horse	Healthy horse
	15	Gelding	Pure Spanish horse	Healthy horse
	13	Gelding	Crossbred	Healthy horse
	14	Mare	Crossbred	Healthy horse
	4	Mare	Pure Spanish horse	Healthy horse

The healthy population (*n* = 6) was composed of horses admitted for castration or routine health check in the Veterinary Teaching Hospital of the University of Extremadura and of privately owned horses from a stable in the province of Almería (Spain) (Table 1). The healthy horses showed no clinical signs of abdominal pain [15] during the physical examination. In addition, the heart rate (HR) and respiratory rate (RR) were within normal limits and they had no haematological or biochemical abnormalities.

### Confirmatory study

The diseased population (*n* = 37), diagnosed as having acute gastrointestinal disease, came from the Veterinary Teaching Hospital of the University of Extremadura between January and October 2018. It was composed of 14 geldings, 14 stallions and 9 mares with 9 ± 4.6 years of age, including 12 Pure Spanish horses, 11 Crossbreds, 4 Warmbloods breeds, 4 Arabians, 3 Lusitanian horses, 2 Thoroughbreds, and 1 Friesian horse. The diagnosis was performed as in the pilot study and included enteritis (*n* = 8), strangulating lesion in the small intestine (*n* = 7), impaction of the pelvic flexure (*n* = 5), large colon displacement (*n* = 4), impaction of the right dorsal colon (*n* = 3), colitis (n = 3), large colon volvulus (*n* = 2), cecal tympany (*n* = 1), gastric perforation (*n* = 1), impaction of the small colon (*n* = 1), gastric ulcer syndrome (*n* = 1) and fecaloma in the small colon (*n* = 1).

The healthy population (*n* = 20) was selected as in the pilot study. It included 8 stallions, 6 females and 6 geldings, with an average age of 9 ± 4.4 years. The breeds included 14 Pure Spanish horses, 4 Crossbreds, 1 Arabian and 1 Thoroughbred.

### Sampling

Saliva samples were obtained in the diseased population within 30 min of arrival at the hospital once horses were in their box stalls. Upon arrival at the hospital, saliva samples were obtained prior to clinical examination or treatment. Saliva was collected by introducing a small sponge (Esponja Marina, La Griega E. Koronis, Madrid, Spain) into the horse’s mouth toward the side of the cheeks, allowing the sponge to be chewed until it was soaked with saliva. The entire procedures took around 1 min. Then, sponge was placed in collection devices (Salivette, Sarstedt, Aktiengesellschaft & Co, Nümbrecht, Germany) and was proceessed as previously reported [16]. Blood samples were collected just after saliva sampling by jugular venipuncture and transferred into lithium-heparin tubes (Li Heparin, Aquisel®, Barcelona, Spain). Horses that yielded plasma samples with gross haemolysis and blood-contaminated saliva samples were excluded from the study. Horses with increased digital pulses or evident lameness were excluded in order to avoid clinical cases combined with laminitis or musculoskeletal problems (Additional file 1). Information about HR, RR and rectal temperature were also obtained from the diseased and healthy populations.

### Plasma and saliva biochemistry profile

The chemistry profile measured in plasma was integrated by:

- Enzymes: AST, ALP, γ-glutamyl transferase (gGT), lipase, amylase, LDH, CK, butyrilcholinesterase (BChE) and ADA.
- Metabolites: creatinine, urea, uric acid, total bilirubin, cholesterol, triglycerides, glucose and lactate.
- Proteins: total proteins, albumin, serum amyloid A (SAA) and Haptoblobin (Hp).
- Minerals: P and total calcium.

These assays were carried out on an automated chemistry analyser (Olympus Diagnostica GmbH AU 600), using commercial kits from Beckman (Beckman Coulter Inc., Fullerton, CA, USA) for all assays with the exception of ADA, which was measured with a Diazyme kit (ADA-D assay kit, Diazyme Laboratories, Poway, CA, USA), and BChE, which was measured according to a previously reported assay [17]. All these assays showed low inter and intra-assay imprecision (< 10%) and high linearity under dilution (*R*^*2*^ > 0.995) in serum.

The same chemistry profile, methods and apparatus were also used in saliva samples, with the exception of two analytes in which the analytical method was different: total proteins, which was evaluated using a commercial colorimetric kit to measure urine and Low-Complexity Region (LCR) proteins (protein in urine and CSF, Spinreact, Spain), and BChE, which was measured following a previously described method [6]. In addition, two new analytes were measured in saliva samples: TEA activity, which was measured using a previously described method [5] in the Olympus chemistry analyser, and cortisol, which was analysed with a solid-phase competitive enzyme-amplified chemiluminescent immunoassay by the immunoassay system (Immulite 1000, Siemens Healthcare Diagnostic, Deerfields, IL) and validated in saliva by Escribano et al. [18]. The analytes in saliva were validated by the researchers’ laboratory, showing an inter- and intra-variability lower than 10% of coefficient of variation (CV) and were linear when applied to serial sample dilutions (*R*^*2*^ = 0.992 ± 0.004) (Additional file 2). SAA and Hp could not be detected in saliva with the assays used in plasma (LZ SAA, Eiken Chemical Co.; and Haptoglobin turbidimetry, Spinreact, Spain, respectively). Therefore, they were excluded from the saliva evaluation. SAA and Hp assays used anti-human serum antibodies. The SAA assay used has been demonstrated to have cross-reactivity and adequate accuracy for SAA measurement in horses’ plasma [19, 20], and the antibodies used in the assay for Hp measurement have cross-reactivity with plasma Hp in horse by western blot (data no showed), similarly to other human antibodies that showed cross-reactivity with Hp of horses [21].

### Severity scale

The severity of the acute abdominal disease was assessed using the systemic inflammatory response syndrome (SIRS) score developed by Roy et al. [22], since it is easy to apply and because it has been demonstrated in a large population to be closely associated with the risk of death, mainly in cases of acute gastrointestinal illness cases. It was calculated as the sum of abnormal results in HR (> 52 beats/min), RR (> 20 breaths /min), white blood cell (WBC) count (above or below 5.0–12.5 × 10^9^/L) and temperature (below or above 37.0–38.5 °C). Horses were considered to have SIRS when the score was equal to or higher than 2 (4-point-score).

### Statistical analysis

To evaluate if there were significant differences in the analytes evaluated in saliva between the healthy and diseased populations in the pilot exploratory study, an unpaired Student’s *t* test (2-tailed) was performed. Previously, the values were transformed by log applying the formula ln x = ln (x + 1) [23], since all showed non-normal distribution when checked using the Shapiro-Wilk test. The analytes that showed significant changes were studied in the confirmatory population using the statistical analysis described above. A stand-alone power program for statistical testing (G-Power) [24] was employed using the means and standard deviations of the selected analytes initially calculated in the pilot exploratory study, to establish the minimum number of individuals necessary to be included in the confirmatory study in each group for reaching a significance level of α = 5% (*P* < 0.05) and a power of 80%. Additionally, a post hoc analysis was performed with the number of individuals used in the confirmatory study to guarantee that the significance level and power required were correctly obtained.

Spearman correlation was performed on the analytes of the confirmatory study, in order to evaluate a correlation between them in saliva and plasma, and between the SIRS score. An *r* value from 0.90 to 1 was considered to have very high correlation, 0.70 to 0.90 high correlation, 0.50 to 0.70 moderate correlation, 0.30 to 0.50 low correlation and less than 0.30 little if any correlation [25]. A receiver operator characteristic (ROC) curve analysis was also performed to obtain the optimal cut-off point to maximise sensitivity and specificity for determining the healthy and diseased population with acute abdominal disease. All statistical analyses were performed using a spreadsheet (Excel 2000, Microsoft Corporation, Redmond, Washington, USA) and the commercial statistics package Graph Pad Prism 6 (GraphPad Software Inc., La Jolla, CA, USA). Values of *P* ≤ 0.05 were selected to indicate significance in all analyses.

## Results

### Differences in salivary analytes between diseased and healthy populations

Medians and interquartile ranges (IQR) from all the analytes evaluated in the pilot exploratory study are shown in Table 2. There were seven analytes that showed a significant increase in horses with acute abdominal disease compared to healthy horses: gGT (showing a 1.8 difference between log means, 95% confidence interval [CI] 0.6–3.0, *P* = 0.008), CK (1.5 difference between log means, 95% CI 0.1–2.9, *P* = 0.04), urea (0.9 difference between log means, 95% CI 0.2–1.6, *P* = 0.02), total bilirubin (0.2 difference between log means, 95% CI 0.1–0.4, *P* = 0.05), total protein (1.0 difference between log means, 95% CI 0.4–1.6, *P* = 0.004), P (0.6 difference between log means, 95% CI 0.2–1.3, *P* = 0.05) and sAA (2.6 difference between log means, 95% CI 1.3–3.8, *P* = 0.001).

**Table 2:** Medians, interquartile ranges (IQR, 25–75%), minimal (Min) and maximal (Max) and values of a biochemical analysis performed in saliva from 6 healthy horses and 6 horses diagnosed with acute abdominal disease

	Healthy horses (*n* = 6)				Diseased horses (*n* = 6)			
	Median	Min	Max	IQC	Median	Min	Max	IQC
AST (IU/L)	101.8	45.5	205.8	47.8–146.3	212.8	28.1	1216.9	68.5–615.7
ALP (IU/L)	58.5	1.0	79.8	30.3–74.4	131.6	48.0	355.7	48.9–198.2
gGT (IU/L)	31.1	2.5	62.8	19.3–51.8	199.5**	55.9	359.0	65.9–331.5
Lipase (IU/L)	49.8	10.0	100.1	13.23–75.3	26.4	6.7	91.6	16.3–76.8
LDH (IU/L)	270.7	0.0	1195.0	41.9–662.3	688.6	42.3	2896.0	43.9–1795.0
CK (IU/L)	11.1	0.0	23.5	2.4–15.4	30.1*	7.0	140.7	13.5–88.9
BChE (nmol/mL/min)	19.2	5.6	31.1	12.1–27.5	18.6	12.5	62.1	13.6–57.7
ADA (IU/L)	14.5	8.2	85.2	12.4–36.2	41.1	11.8	216.3	26.9–111.6
Creatinine (μmol/L)	28.3	8.8	285.5	13.3–114.0	33.6	0.9	106.1	3.5–64.53
Urea (mmol/L)	7.9	4.1	10.4	5.4–9.7	18.8*	8.2	44.9	9.9–33.7
Uric acid (μmol/L)	59.5	51.8	103.5	52.3–86.8	38.7	0.6	88.6	10.7–71.4
Total bilirubin (μmol/L)	2.9	1.0	10.3	1.7–6.2	7.5*	3.6	13.5	4.3–10.4
Cholesterol (μmol/L)	35.4	0.00	249.8	0.0–169.7	93.4	0.0	263.8	0.00–187.0
Triglycerides (mmol/L)	0.34	0.05	0.68	0.06–0.67	0.21	0.02	3.64	0.04–1.17
Glucose (mmol/L)	0.54	0.01	1.80	0.13–1.05	0.51	0.00	2.99	0.16–2.24
Lactate (mmol/L)	1.73	0.02	3.44	0.42–2.70	0.39	0.01	20.91	0.09–9.32
Total protein (g/L)	1.65	0.96	4.23	1.01–2.72	5.77**	3.47	6.18	3.53–5.99
Albumin (g/L)	0.5	0.1	0.9	0.3–0.8	0.8	0.1	2.6	0.4–1.5
Phosphorus (mmol/L)	0.26	0.04	0.48	0.12–0.34	0.59*	1.20	2.04	0.24–1.72
Calcium (mmol/L)	4.7	4.2	7.0	4.2–7.0	6.4	4.1	9.4	4.8–9.0
sAA (IU/L)	13.5	1.0	23.1	4.2–20.3	109.3**	43.7	544.3	51.7–446.5
Cortisol (μg/dL)	0.93	0.56	1.12	0.79–1.05	0.91	0.6	1.08	0.60–1.02
TEA (IU/L)	141.7	77	165.5	120.7–159.4	133	39.8	184.9	44.2–133.0

*AST* aspartate aminotransferase, *ALP* alkaline phosphatase, *gGT* γ-glutamyl transferase, *LDH* lactate dehydrogenase, *CK* creatine kinase, *BChE* butyrilcholinesterase, *ADA* adenosine deaminase, *sAA* alpha-amylase salivary, *TEA* total esterase

Asterisk indicates statistically significant differences between groups (*: *P* < 0.05; **: *P* < 0.01)

**Fig. 1:**
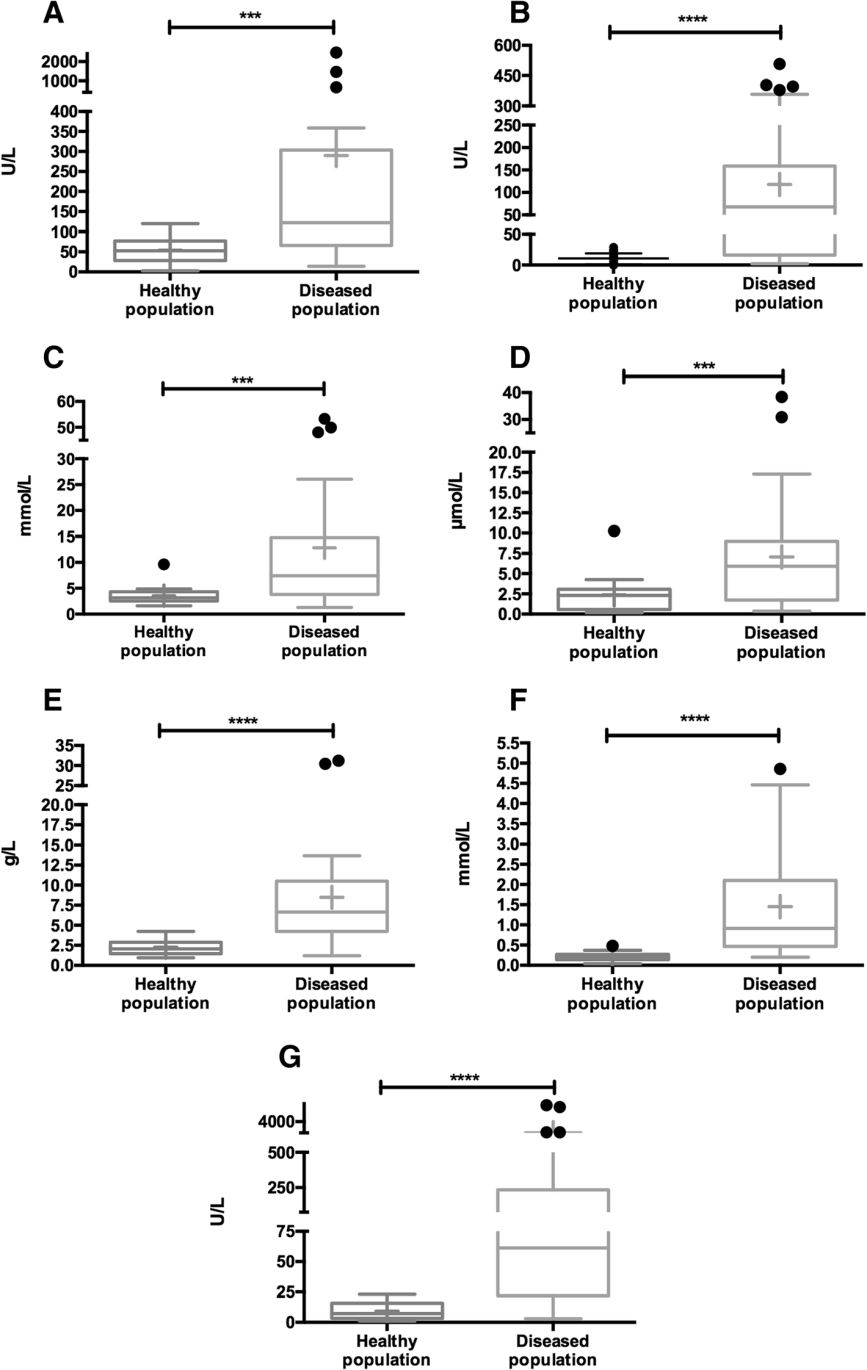
Results of γ-glutamyl transferase (gGT) (**a**), creatine kinase (CK) (**b**), urea (**c**), total bilirubin (**d**), total protein (**e**), phosphorus (**f**) and salivary alpha-amylase (sAA) (**g**) in saliva of a healthy horses population (*n* = 20) and a diseased horses population (*n* = 37) with acute abdominal disease. The plot shows median (line within box), 25th–75th percentiles (box), 5th and 95th percentiles (whiskers) and outliers (•). The cross inside the box shows the mean. Asterisk indicates statistically significant differences between groups (***: *P* < 0.001; ****: *P* < 0.0001)

The power study calculated with the data from the pilot exploratory study showed that a minimum number of 11 healthy horses and 21 horses with acute abdominal disease would be needed to get appropriate results for the selected analytes. The post hoc analysis performed in the confirmatory study computed a power 96.4 ± 0.1% for the gGT, CK, urea, total bilirubin, total protein, P and sAA results. When these analytes were evaluated in the study made in the larger population, horses with acute abdominal disease had values statistically higher than the healthy ones for all the analytes (Fig. 1).

The optimal cut-off point in saliva for predicting disease due to acute abdominal disease in the selected analytes was gGT ≥ 82.7 IU/L (AUC = 0.79; 95% CI: 0.67–0.92; sensitivity 70% [51–85]; specificity 80% [56–94]), CK ≥ 30.1 IU/L (AUC = 0.85; 95% CI: 0.74–0.95; sensitivity 64% [45–81]; specificity 100 [83–100]), urea ≥5.3 mmol/L (AUC = 0.79; 95% CI: 0.67–0.92; sensitivity 66% [46–82]; specificity 95% [75–100]), total bilirubin ≥4.4 μmol/L (AUC = 0.73; 95% CI: 0.60–0.86; sensitivity 56% [38–72]; specificity 95% [75–100]), total proteins ≥4.24 g/L (AUC = 0.95; 95% CI: 0.90–1.01; sensitivity 77% [60–90]; specificity 100% [83–100]), *P* ≥ 0.50 mmol/L (AUC = 0.94; 95% CI: 0.89–1.00; sensitivity 75% [58–88]; specificity 100% [83–100]) and sAA ≥ 23.3 IU/L (AUC = 0.92; 95% CI: 0.86–1.00; sensitivity 73% [56–86]; specificity 100% [83–100]).

### Correlations

The correlations between the different analytes selected in saliva, and between these analytes in saliva and plasma are shown in Table 3. High correlations in saliva were observed between gGT and urea (r = 0.70, *P* < 0.001), CK and phosphorus (r = 0.71, *P* < 0.001) and total proteins and P (r = 0.75, *P* < 0.001). Moderate correlations in saliva were observed between gGT and total proteins (r = 0.67, *P* < 0.001), P (r = 0.61, *P* < 0.001) and CK (r = 0.68, *P* < 0.001); between CK and total proteins (r = 0.69, *P* < 0.001), urea (r = 0.66, *P* < 0.001), total bilirubin (r = 0.57, *P* < 0.001) and gGT (r = 0.68, *P* < 0.001); between urea and total proteins (r = 0.61, *P* < 0.001) and P (r = 0.52, *P* < 0.001); and between total proteins and sAA (r = 0.61, *P* < 0.001).

**Table 3:** Spearman correlation coefficients between gGT, CK, urea, total bilirubin, total protein, phosphorus and sAA in saliva and plasma in healthy (*n* = 20) and diseased horses (*n* = 37) with acute abdominal disease

		Saliva						
		gGT (IU/L)	CK (IU/L)	Urea (mmol/L)	Total bilirubin (μmol/L)	Total proteins (g/L)	Phosphorus (mmol/L)	sAA (IU/L)
gGT	saliva		0.68***	0.70***	0.24	0.67***	0.61***	0.26
(IU/L)	plasma	0.24	0.15	0.08	0.03	0.41*	0.36*	0.09
CK	saliva	0.68***		0.66***	0.57***	0.69***	0.71***	0.45**
(IU/L)	plasma	0.22	0.34*	0.36*	0.3	0.31	0.34*	0.55***
Urea	saliva	0.70***	0.66***		0.34*	0.61***	0.52***	0.37**
(mmol/L)	plasma	0.19	0.24	0.49**	0.36*	0.35*	0.3	0.26
Total bilirubin	saliva	0.24	0.57***	0.34*		0.36**	0.37**	0.49***
(μmol/L)	plasma	0.19	0.3	0.08	0.37*	0.51***	0.60***	0.39*
Total proteins	saliva	0.67***	0.69***	0.61***	0.36**		0.75***	0.61***
(g/L)	plasma	0.32*	0.34*	0.35*	0.18	0.34*	0.29	0.26
Phosphorus	saliva	0.61***	0.71***	0.52***	0.37**	0.75***		0.48***
(mmol/L)	plasma	0.06	−0.08	0.07	−0.16	0.18	0.1	0.19
Amylase	saliva	0.26	0.45**	0.37**	0.49***	0.61***	0.48***	
(IU/L)	plasma	−0.30	−0.24	−0.45*	−0.02	0.26	0.14	0.24

Asterisk indicates statistically significant difference between comparisons

*gGT* γ-glutamyl transferase, *CK* creatine kinase, *sAA* salivary alpha-amylase

Asterisk indicates statistically significant correlations (*: *P* < 0.05; **: *P* < 0.01; ***: *P* < 0.001)

Positive correlations between saliva and plasma were observed in CK (*r* = 0.34, *P* = 0.05), urea (*r* = 0.49, *P* = 0.002), total bilirubin (*r* = 0.37, *P* = 0.02) and total proteins (*r* = 0.34, *P* = 0.03). The SIRS score correlated in saliva with CK (*r* = 0.40, *P* = 0.03), total bilirubin (*r* = 0.41, *P* = 0.02), total proteins (*r* = 0.49, *P* = 0.003), P (*r* = 0.50, *P* = 0.002) and sAA (*r* = 0.35, *P* = 0.05).

## Discussion

This report describes for the first time that a profile integrated by various analytes that are used in routine serum biochemistry analysis can be measured in saliva in horses, and that some of these analytes can change in situations of acute abdominal disease.

In this report, we followed a similar approach that is made in proteomics studies when changes in a profile of proteins are studied in a clinical situation [26–28]. First, an exploratory study in a small population was performed in order to detect possible analytes that can change in the situation being studied, in our case acute abdominal disease. This population, as described in previous studies, comprised horses with different ages, sexes, and diagnoses in order to be representative of the different clinical conditions that can appear in this disease [14, 29]. Then, the analytes showing changes were validated in a larger population.

Seven analytes were detected as being raised in saliva of horses with acute abdominal disease. Two of them were gGT and total bilirubin, which in serum are associated with liver dysfunction [30, 31]. Elevations in serum activity and concentration of both analytes have been associated with several causes of colic in horses, including cholelithiasis, infectious colitis, large colon displacement, ulcerative duodenitis, proximal enteritis and neoplasia [32]. Total bilirubin in saliva correlated moderately with plasma; therefore it could be postulated that part of the bilirubin that appears in saliva comes from plasma. In addition, total bilirubin was correlated moderately with the SIRS score, so it could be associated with the severity of the disease.

CK, which in serum is associated with muscle damage [33], was raised in our study in the saliva of diseased horses. Increases in CK in saliva in situations of muscle damage have been described in dogs [34] and humans [7] and, therefore, the high values of CK in saliva of horses with acute abdominal disease could indicate the existence of muscle damage, which has been described as occurring in this disease by ischemic injuries [35, 36]. One source of saliva CK could be the CK from plasma, since significant correlations were found between CK in plasma and saliva in our study, similarly to that reported in dogs [34]. In our study, CK in saliva had a significant although low correlation with SIRS status and therefore it could be an indicator of the severity of the process.

Increases in serum urea with normal values of creatinine are associated with reduced hydration status [37] or bleeding in the gastrointestinal tract [38]. Therefore, it could be postulated that similar causes could produce the higher salivary urea found in our study in horses with acute abdominal disease. A moderate correlation between urea in saliva and plasma was observed. This correlation has also been reported in dogs with chronic kidney disease [39]. The higher concentration of total proteins observed in saliva in horses with acute abdominal disease could also indicate reduced hydration status, as it has previously been described in serum [40].

P in serum has been considered a marker of disease severity and likelihood of mortality in hospitalised foals [41]. Although in this study P in saliva did not correlate with P in plasma, it correlated in saliva with the SIRS score. Further studies should be performed in order to clarify the undergoing mechanisms of P in saliva.

The increases in sAA activity found in our study on diseased horses were in agreement with other studies performed on horses with acute abdominal disease [3, 16]. In addition, a low but significant correlation of sAA with SIRS status was observed in our study, as previously reported [3, 16]. The lack of correlation with amylase in plasma confirms that, as humans, the sAA that appears in saliva is secreted by the salivary glands [23].

From all the analytes that showed differences between the horses with acute abdominal disease and the healthy ones, total proteins, P and sAA showed sensitivities higher than 70% at their optimal cut-off points and a specificity of 100% in detecting acute abdominal disease. Ideally, further studies in which these analytes are evaluated in other diseases should be performed, in order to evaluate their specificity not only in the case of healthy individuals but also in individuals with diseases other than acute abdominal disease.

The power analysis guaranteed adequate statistical analysis for evaluating differences between healthy and diseased horses in the confirmatory population. However, it would be of interest to make future studies in a larger population to evaluate if changes in analytes could be related with outcome (survivors vs. non-survivors), diagnosis (ischaemic/strangulating vs. non-ischaemic/non-strangulating) and treatment needed (medical treatment vs. surgery). In addition, it would have been interesting to include an analysis of electrolytes in saliva in this study, which was not possible due to volume constraints.

## Conclusions

A panel of 23 analytes can be measured in saliva in horses, and from these analytes, gGT, CK, urea, total bilirubin, total protein, phosphorus and sAA can be raised in horses with acute abdominal disease. Further studies should be made to evaluate and refine the possible application of these analytes in clinical situations.

## Additional files

Additional file 1: Flow chart describing horses included and excluded in the diseased populations. Description of horses included or excluded from the study in the diseased populations. (PDF 225 kb)

Additional file 2: Linearity under dilution in saliva of aspartate aminotransferase (AST), alkaline phosphatase (ALP), γ-glutamyl transferase (gGT), lipase, lactate dehydrogenase (LDH), creatine kinase (CK), butyrilcholinesterase (BChE), adenosine deaminase (ADA), creatinin, urea, uric acid, total bilirubin, cholesterol, triglycerides, glucose concentration, total proteins, albumin, lactate, phosphorus (P), calcium and total esterase (TEA). Linearity under dilution study in three pools of saliva from two specimen of saliva each. The ‘x’ expressed activity or concentration measured and ‘y’ expected level at the particular dilution. *R*^*2*^ = coefficient of determination of linear correlation. (PDF 2190 kb)

## Data Availability

The datasets generated and/or analysed during the current study are not publicly available due to legal reasons but are available from the corresponding author on reasonable request.

## Abbreviations

ADA: Adenosine deaminase
ALP: Alkaline phosphatase
ALT: Alanine aminotransferase
AST: Aspartate aminotransferase
BChE: Butyrilcholinesterase
ChE: Cholinesterase
CI: Confidence interval
CK: Creatine kinase
CV: Coefficient of variation
gGT: γ-glutamyl transferase
Hp: Haptoblobin
HR: Heart rate
IQR: Interquartile ranges
LCR: Low-complexity region
LDH: Lactate dehydrogenase
P: Phosphorus
ROC: Receiver operator characteristic
RR: Respiratory rate
sAA: Salivary apha-amylase
SAA: Serum amyloid A
SIRS: Systemic inflammatory response syndrome
TEA: Total esterase activity
WBC: White blood cells
