# Supplementary figures and images for: Changes in saliva analytes in equine acute abdominal disease: a sialochemistry approach
Source: BMC Vet Res. 2019 Jun 6;15:187. doi: 10.1186/s12917-019-1933-6 (PMC6554884; doi:10.1186/s12917-019-1933-6)

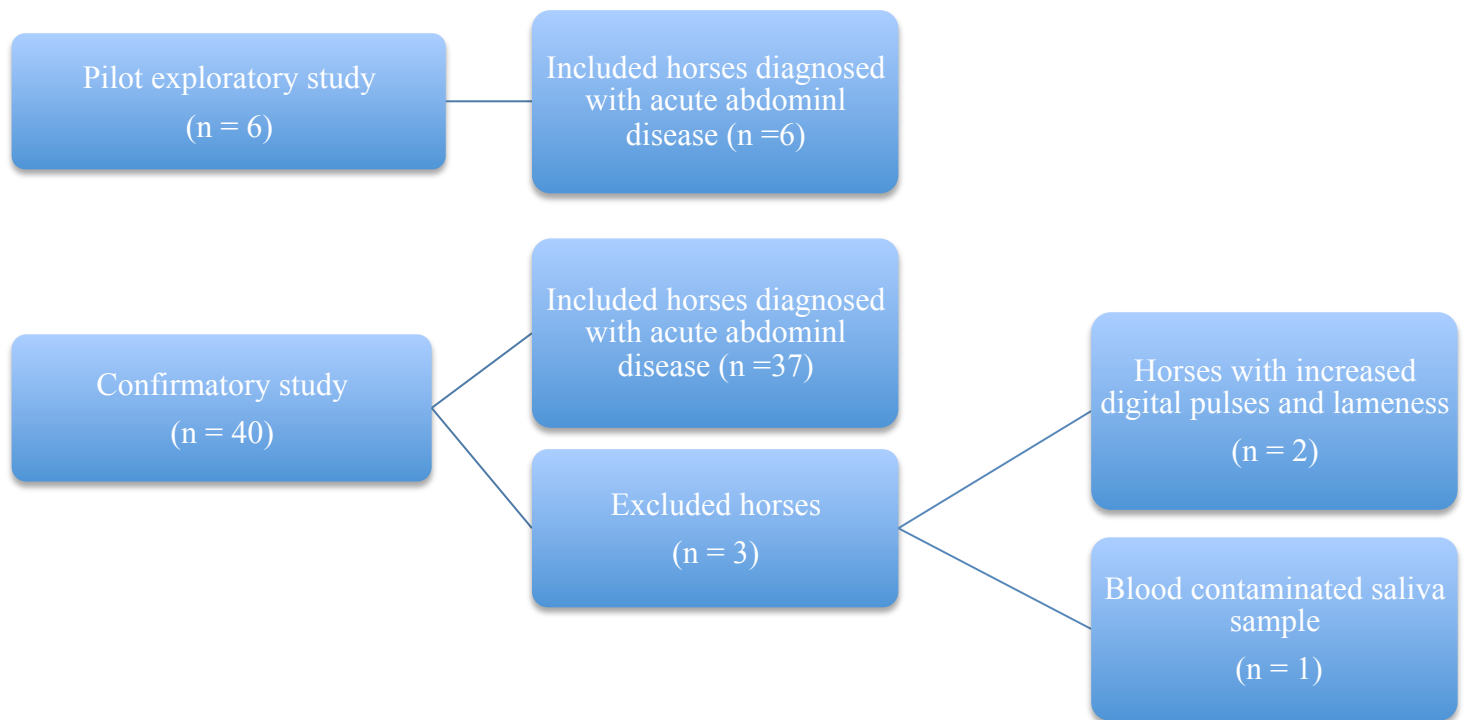

Supplement: Supplementary file 1 — Flow chart describing horses included and excluded in the diseased populations. Description of horses included or excluded from the study in the diseased populations. (PDF 225 kb) [file 12917_2019_1933_MOESM1_ESM.pdf]

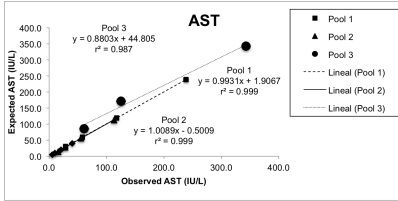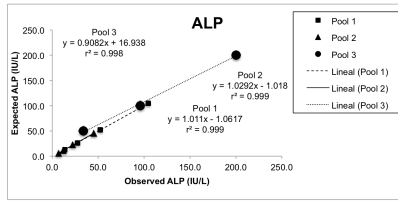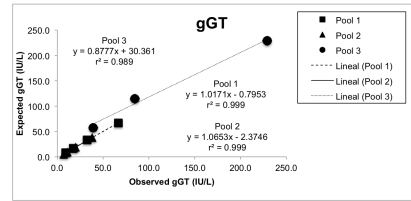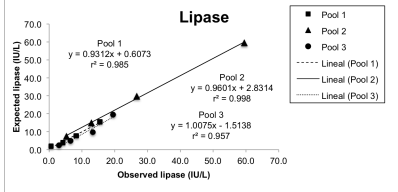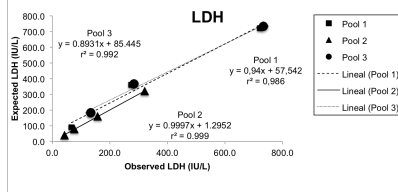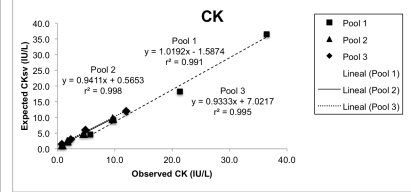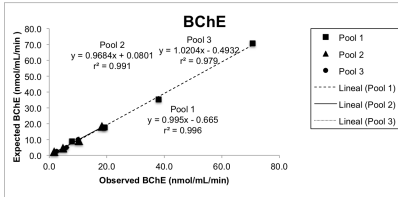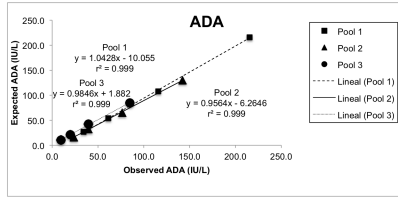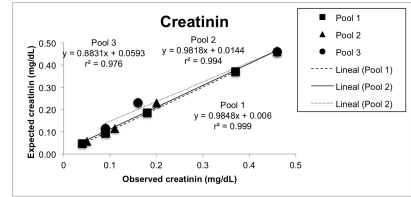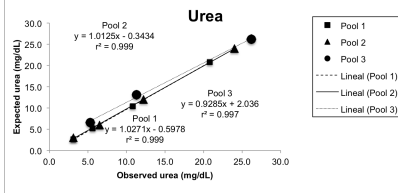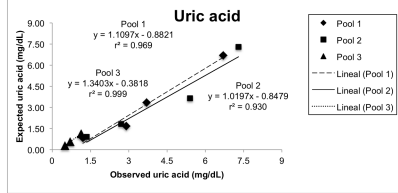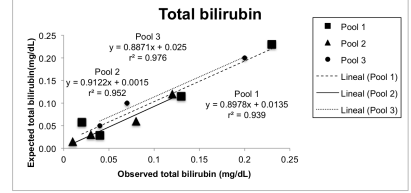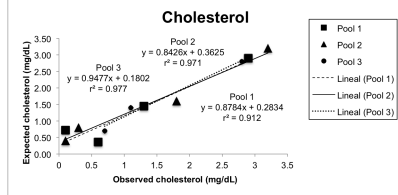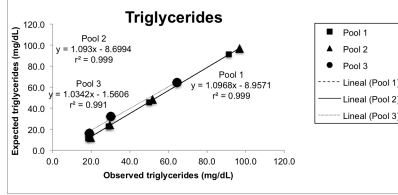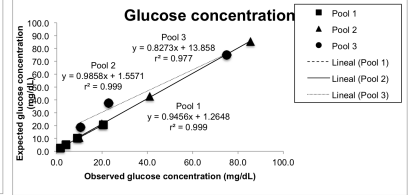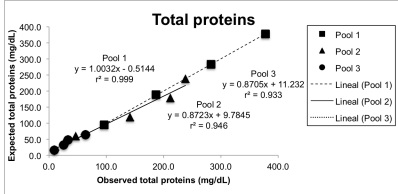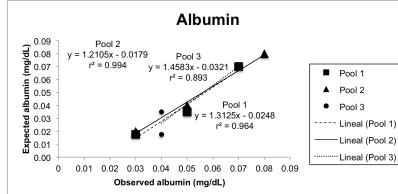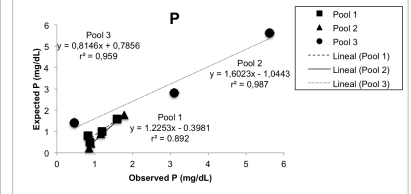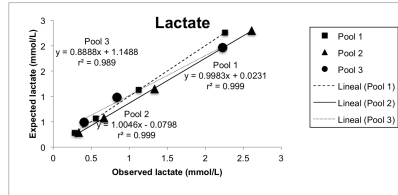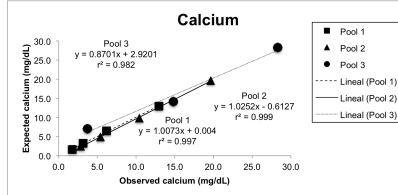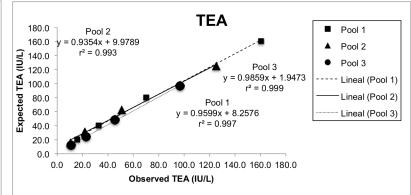

Supplement: Supplementary file 2 — Linearity under dilution in saliva of aspartate aminotransferase (AST), alkaline phosphatase (ALP), γ-glutamyl transferase (gGT), lipase, lactate dehydrogenase (LDH), creatine kinase (CK), butyrilcholinesterase (BChE), adenosine deaminase (ADA), creatinin, urea, uric acid, total bilirubin, cholesterol, triglycerides, glucose concentration, total proteins, albumin, lactate, phosphorus (P), calcium and total esterase (TEA). Linearity under dilution study in three pools of saliva from two specimen of saliva each. The ‘x’ expressed activity or concentration measured and ‘y’ expected level at the particular dilution. R2 = coefficient of determination of linear correlation. (PDF 2190 kb) [file 12917_2019_1933_MOESM2_ESM.pdf]
